# Supplementary material for: Difference in racket head trajectory and muscle activity between the standard volley and the drop volley in tennis
Source: PLoS One. 2021 Sep 14;16(9):e0257295. doi: 10.1371/journal.pone.0257295 (PMC8439447; doi:10.1371/journal.pone.0257295)
Supplement: Attachment — Submitted filename: Responses to reviewers .docx [file pone.0257295.s002.docx]

Reviewer #1: Authors answered positively to my previous comments and included the purposed changes in the manuscript.
I thank the authors for having responded positively to my comments. Congratulations for the great work.

A. Thank you. We deeply appreciate your help. By modifying the manuscript according to your suggestions and advice, the manuscript has become much better.

Reviewer #2: The authors have completed substantial revisions to this manuscript and have addressed several of the significant concerns I identified in my first review. The clarity of writing and grammar are much improved and the methods are much clearer.
The most important concerns I identified previously related to the appropriateness of the analysis methods when examining time series data and the effects of the various averaging processes which could compound the analysis. I suggested that these concerns may be addressed using established methods for time series analysis such as functional data analysis or statistical parametric mapping (random field theory). The authors elected to revise the analysis but only changed the time intervals for their analysis. The difficulties of averaging data from within subjects (trials and interval averaging) and pooling this with between subjects data to derive a global within and between subjects averages still remains. Furthermore the authors did not use an established method for time series/ curve data analysis to examine the data and this presents further problems.
I believe the authors may have some valuable data that could provide some useful insights but we cannot really be sure of this until the data are appropriately analysed. This was the major concern I raised and I do not believe it has been adequately addressed.

A. We appreciate the valuable suggestion. According to your strong suggestion, we utilized statistical parametric mapping (SPM) for a data analysis of the EMGs. The trajectory of the racket head was also analyzed with SPM. As for the EMGs, SPM analysis basically showed the same tendencies of temporal changes. That is, there was no decrease in the EMGs toward impact, meaning that muscle relaxation did not occur in the drop volley, our main conclusion. Accordingly, we made appropriate changes in Figs. 4, 7, and 8, and related text. We are confident that the new manuscript will present the results more clearly than did the previous version. We really appreciate the reviewer’s suggestion that we utilize an elegant, appropriate method.
